# Modern contraceptive use among sexually active men in Uganda: does discussion with a health worker matter?

**DOI:** 10.1186/1471-2458-14-286

**Published:** 2014-03-28

**Authors:** Allen Kabagenyi, Patricia Ndugga, Stephen Ojiambo Wandera, Betty Kwagala

**Affiliations:** 1 Department of Population Studies, School of Statistics and Planning, College of Business and Management Sciences, Makerere University, Kampala, Uganda; 2 Centre for Population and Statistics, Makerere University, Kampala, Uganda

**Keywords:** Male involvement, Family planning, Health worker, Contraception, Discussion, Uganda

## Abstract

**Background:**

Family planning programs have recently undergone a fundamental shift from being focused on women only to focusing on men individually, or on both partners. However, contraceptive use among married men has remained low in most high-fertility countries including Uganda. Men’s role in reproductive decision-making remains an important and neglected part of understanding fertility control both in high-income and low-income countries. This study examines whether discussion of family planning with a health worker is a critical determinant of modern contraceptive use by sexually active men, and men’s reporting of partner contraceptive use.

**Methods:**

The study used data from the 2011 Uganda Demographic and Health Survey comprising 2,295 men aged 15–54 years. Specifically, analyses are based on 1755 men who were sexually active 12 months prior to the study. Descriptive statistics, Pearson’s chi-square test, and logistic regression were used to identify factors that influenced modern contraceptive use among sexually active men in Uganda.

**Results:**

Findings indicated that discussion of family planning with a health worker (OR =1.85; 95% CI: 1.29–2.66), region (OR = 0.41; 95% CI: 0.21–0.77), education (OR =2.13; 95% CI: 1.01–4.47), wealth index: richer (OR = 2.52; 95% CI: 1.58–4.01), richest (OR = 2.47; 95% CI: 1.44–4.22), surviving children (OR = 2.04; 95% CI:1.16–3.59) and fertility preference (OR = 3.50; 95% CI: 1.28–9.61) were most significantly associated with modern contraceptive use among men.

**Conclusions:**

The centrality of the role of discussion with health workers in predicting men’s participation in family planning matters may necessitate creation of opportunities for their further engagement at health facilities as well as community levels. Men’s discussion of family planning with health workers was significantly associated with modern contraceptive use. Thus, creating opportunities through which men interact with health workers, for instance during consultations, may improve contraceptive use among couples.

## Background

Like many sub-Saharan countries, Uganda still grapples with a low contraceptive prevalence rate (CPR) (30%) and a high fertility rate (TFR) (6.2), the latter having stalled for the last three decades [1]. This has contributed significantly to a high population growth rate of over 3.2% that puts pressure on already meager resources and poses a serious challenge to service provision. Specific and well elaborated strategies to improve reproductive health and address the population challenge are required [2]. Family planning (FP) services have been promoted as critical in giving couples the freedom to space and plan the number of children they wish, but also contributing to the health and overall quality of life of the population [3].

Men, particularly in patriarchal contexts, play a central role in influencing fertility decisions [4,5]. However, male involvement in family planning remains limited despite the 1994 International Conference on Population and Development in Cairo, which emphasized the need for men’s involvement in sexual and reproductive health issues [6,7]. Likewise, research investigating factors associated with couples’ contraceptive use has neglected men’s central role [8-10], which has ultimately resulted in reinforcing the idea of family planning as women’s responsibility, leaving little or no role for men [11-13].

It has been observed [14-16] that men, particularly in agrarian subsistence economies, prefer large numbers of children both as a source of labor and economic gain, and as a source of prestige. Given men’s role as decision makers, such perceptions are believed to deter men’s and couples’ utilization of contraceptives [16,17]. A husband’s approval of the use of contraception is crucial for successful family planning programs [18,19]. A study in Kenya illustrated that husbands had absolute decision-making power and the ability to effect compliance or submission from their wives [15]. This was also observed in Zimbabwe and Ghana [20,21].

A challenge confronting the redirection of family planning services toward greater male involvement and couples’ collective decision-making is how to effectively enlist the participation of men [22,23].

In sub-Saharan Africa, the influence of discussing contraception among couples has been investigated to some extent and has been highlighted as an avenue that triggers and enhances contraceptive use [19,24,25]. For instance, among women residing in six countries of sub-Saharan Africa, those who reported frequent communication with their partner about contraception had increased odds of using a family planning method over those who reported never discussing the topic [7].

Provider factors are equally important in influencing contraceptive use. For instance, the facilitation of making informed choices in family planning is associated with better satisfaction and compliance with the method. One consequence is fewer failures [3,26]. Health care providers are an important source of information about family planning and their opinions about specific methods can also influence couple choices [27]. To promote use of modern contraceptives in formerly pro-natalist countries like Albania, communication campaigns that involved training health care providers were used to assure relevant populations about the safety of modern contraceptives [28].

In other instances, engaging volunteers to use interpersonal communication through household visits and group discussions has been used to disseminate accurate information on family planning methods in countries such as Guinea and Nepal [29,30].

Whereas health provider factors have the potential to influence contraceptive men. The client– provider interaction provides opportunities use, few studies have examined the factors associated with modern contraceptive use (MCU) among men in the developing world, and particularly discussion of family planning with a health worker. No study has analyzed the role of health workers’ discussion of FP with men in effecting modern contraceptive use among Ugandan for provision of accurate information, allaying fears and misconceptions and, in the case of couples, joint decision-making regarding the different family planning options [8,27].

Using the 2011 Uganda Demographic and Health Survey (UDHS) dataset, this study examined the influence of discussing family planning with health workers on modern contraceptive use (MCU) among sexually active men, and their reports of partner contraceptive use. We also examined a series of individual level factors including the desire to have other children and the number of children surviving, to measure how these may ultimately influence men’s contraceptive use.

## Methods

### Data source

This paper is based on the Uganda Demographic and Health Survey (UDHS) that was conducted in 2011. Authorization to use this data (accessed from the MEASURE DHS website) was obtained upon providing a brief description of our study. Two-stage cluster sampling was used to generate a representative sample of 2,573 men aged 15–54. Informed consent for participation in the study was acquired from the male respondents. The first stage involved selecting the clusters while the second stage selected the households in each cluster. Stratification of urban and rural areas was taken into account. A sample of 404 primary sampling units (PSUs) was covered and proportionally allocated among the ten sub-national regions. Details on the sampling procedure are described elsewhere [1].

### Study sample

From a sample of 2,573 men, 1,755 men aged 15–54 who were sexually active in the last 12 months were extracted for further analyses.

### Variables

In the DHS men’s questionnaire, respondents were asked if they or their partner’s had used any method to avoid or prevent a pregnancy the last time they had sex, and the method used (see Additional file 1). The coding categories for current contraceptive method use included: not using, pills, intra-uterine device (IUD), injections, condoms, female sterilization, periodic abstinence, withdrawal, implants/Norplant, lactational amenorrhea (LAM), female condoms, foam or jelly [1].

From these categories, modern contraceptive methods included male methods (male condoms and male sterilization) and female methods (pills, female condoms, injections, the IUD, female sterilization and foam or jelly [31].

We generated a dependent variable (MCU) and coded it as a binary outcome: 1 for men’s reported use of contraception and men's reportage of their partner’s use of contraception, and 0 for nonuse of the same. It is possible that some women use contraceptives without their husband’s knowledge. However, in this context men/husbands were aware and reported their partner/wives’ contraceptive use, suggestive of partnership in contraceptive use.

The independent variables in the study were: age, marital status, residence, region, education level, wealth index and employment status. Intermediate variables included: respondents’ interaction with a health worker, number of living children (children surviving) and fertility preference. In addition attitudinal statements were included in the analyses. These were “women who use contraception become promiscuous” and “contraception is a woman’s business”. Whether participants had consulted with a health worker was obtained from the question “In the last few months, have you discussed family planning with a health worker or health professional?”

### Data analysis

Statistical analyses were conducted at three levels. First, descriptive statistics were performed for both modern contraceptive use and men’s demographic and socioeconomic characteristics. Second, cross tabulations with χ^2^ tests were run to determine the association between modern contraceptive use and men’s sociodemographics. The variables that were significant at 95% were included in the logistic regression models.

Third, binary logistic regression models were fit to predict modern contraceptive use while controlling for independent variables at different stages. Regression diagnostics were performed by testing for the models’ goodness-of-fit and link tests commands in Stata 12.1 (College Station, Tx, USA) [32]. All the analyses were weighted using the *svy* command in Stata to control for the differences in sampling probabilities [33].

## Results

### Descriptive statistics of the respondents

Results presented in Table 1 show that the majority (85%) of the respondents were age 35 and under. About eight in ten respondents (79%) resided in rural areas and few (14%) were from the western region. Seven out of ten (76%) respondents were currently in union. More than half (60%) of the respondents had attained primary education by the time of the survey. Almost all (96%) of the men were currently working in the 12 months preceding the survey.

**Table 1:** Socioeconomic, demographic and behavioral characteristics of respondents

**Variables**	**Percent (%)**	**Frequency (N)**
**Age groups**		
15-24	23.0	404
25-34	36.9	650
35-44	25.3	446
45 & above	14.8	261
**Residence**		
Rural	79.3	1398
Urban	20.7	364
**Region**		
Kampala	10.2	179
Central 1	10.0	176
Central 2	11.6	205
East central	11.1	196
Eastern	13.0	230
North	9.5	168
Karamoja	3.0	53
West-Nile	5.8	103
Western	13.8	243
Southwest	11.9	209
**In union**		
Never	19.1	336
Currently	75.6	1332
Formerly	5.3	94
**Education level**		
None	5.3	94
Primary	59.5	1049
Secondary or higher	35.1	619
**Wealth index**		
Poorest	17.0	300
Poorer	18.7	329
Middle	18.7	329
Richer	21.7	383
Richest	23.9	421
**Currently working**		
No	4.4	77
Yes	95.6	1685
**Children surviving**		
None	22.9	403
1–4	41.0	723
5+	36.1	636
**Fertility preference**		
Have another	47.4	835
Undecided	2.0	36
No more	24.7	435
No partner/infertile/sterilized	25.9	456
**Total**	**100.0**	**1762**
**Contraception is a woman’s business**		
Disagree	81.7	1407
Agree	18.3	316
**Women who use contraception become promiscuous**		
Disagree	59.9	1055
Agree	32.2	568
Don’t know	7.9	139
**Discussed family planning with health worker**		
No	86.2	1519
Yes	13.8	243
**Used modern contraceptives to prevent pregnancy**		
No	67.4	1188
Yes	32.6	574
**Current family planning method type**		
Not using	62.6	1103
Condom – male	17.0	300
Injections	8.2	144
Pill	3.2	56
IUD	0.3	6
Sterilization - female	1.7	29
Implant	1.5	27
Condom – female	0.1	2
Jelly	0.1	1
Traditional methods	5.4	95
**Total**	**100.0**	**1762**

Four in every ten men (41%) had one to four surviving children and almost half the respondents (47%) wished to have another child. Most (82%) of the men disagreed with the statement that “contraception is a woman’s business”. More than half (60%) disagreed with the statement that “women who used contraceptives become promiscuous”. Few men (14%) had discussed family planning with a health worker. A third (33%) of the men reported that they or their partners were using modern contraceptives to prevent pregnancy.

Table 2 shows Pearson’s chi-square tests of the association between modern contraceptive use and men’s sociodemographic factors. Age, residence, region, marital status, education level attained, working status, number of children surviving, and fertility preference were significantly associated with modern contraceptive use (p < 0.001).

**Table 2:** Percent distribution of sexually active men by utilization of modern contraceptive methods in Uganda

**Used modern contraceptives to prevent pregnancy**				
**Variables**	**No (%)**	**Yes (%)**	**Total**	**p-value**
**Age groups**				**0.000**
15-24	56.0	44.0	404	
25-34	69.5	30.5	650	
35-44	72.3	27.7	446	
45+	71.3	28.7	261	
**Residence**				**0.000**
Rural	70.7	29.3	1398	
Urban	54.6	45.4	364	
**Region**				**0.000**
Kampala	51.3	48.7	179	
Central 1	67.9	32.1	176	
Central 2	55.8	44.2	205	
East central	68.2	31.8	196	
Eastern	72.6	27.4	230	
North	65.5	34.5	168	
Karamoja	75.9	24.1	53	
West-Nile	77.1	22.9	103	
Western	66.4	33.6	243	
Southwest	81.3	18.7	209	
**In union**				**0.000**
Never	44.9	55.1	336	
Currently	74.5	25.5	1332	
Formerly	47.3	52.7	94	
**Education level**				**0.000**
None	84.7	15.3	94	
Primary	71.1	28.9	1049	
Secondary or higher	58.4	41.6	619	
**Wealth index**				**0.000**
Poorest	79.6	20.4	300	
Poorer	76.8	23.2	329	
Middle	72.1	27.9	329	
Richer	58.8	41.2	383	
Richest	55.5	44.5	421	
**Currently working**				**0.878**
No	66.5	33.5	77	
Yes	67.4	32.6	1685	
**Children surviving**	53.7	46.3	403	**0.000**
None				
1-4	68.9	31.1	723	
5+	74.4	25.6	636	
**Fertility preference**	76.1	23.9	835	**0.000**
Have another				
Undecided	75.4	24.6	36	
No more	72.8	27.2	435	
No partner/infertile/sterilized	45.7	54.3	456	
**Total**	**67.4**	**32.6**	**1762**	
**Contraception is a woman’s business**				**0.631**
Disagree	67.4	32.6	1407	
Agree	65.8	34.2	316	
**Women who use contraception become promiscuous 0.722**				
Disagree	67.1	32.9	1055	
Agree	67.2	32.8	568	
Don’t know	70.9	29.1	139	
**Discussed family planning with health worker**				**0.002**
No	69.0	31.0	1519	
Yes	57.6	42.4	243	
**Total**	**67.4**	**32.6**	**1762**	

### Multivariate results

Results of the logistic regression of MCU and selected background factors are presented in Table 3. Three models are presented: Model 1 (included discussion of family planning with health worker); Model 2 (adjusted for demographic factors); and Model 3 (adjusted for sociodemographics).

**Table 3:** Likelihood estimates of using modern methods of contraception among sexually active men in Uganda

	**Model (1)**		**Model (2)**		**Model (3)**	
**Variables**	**OR**	**95% [CI]**	**OR**	**95% [CI]**	**OR**	**95% [CI]**
**Discussed family planning with health worker**						
No	1.000	[1.19-2.26]	1.000	[1.23-2.47]	1.000	[1.29-2.66]
Yes	1.640**		1.744**		1.854***	
**Age groups**						
15-24			1.000		1.000	
25-34			0.557^***^	[0.41-0.76]	1.147	[0.73-1.81]
35-44			0.501^***^	[0.36-0.70]	1.278	[0.74-2.20]
45+			0.532**	[0.35-0.80]	1.212	[0.64-2.29]
**Residence**						
Rural			1.000		1.000	
Urban			1.555**	[1.11-2.17]	0.960	[0.64-1.43]
**Region**						
Kampala			1.000		1.000	
Central 1			0.806	[0.44-1.47]	0.819	[0.44-1.51]
Central 2			1.228	[0.70-2.17]	1.347	[0.74-2.45]
East Central			0.713	[0.41-1.24]	0.797	[0.45-1.42]
Eastern			0.630	[0.35-1.14]	1.129	[0.61-2.11]
North			0.902	[0.50-1.64]	1.301	[0.69-2.47]
Karamoja			0.564	[0.20-1.59]	1.202	[0.36-3.97]
West Nile			0.503*	[0.28-0.92]	0.826	[0.43-1.57]
Western			0.810	[0.46-1.42]	0.915	[0.51-1.63]
Southwest			0.391**	[0.21-0.72]	0.406**	[0.21-0.77]
**Marital status**						
Never married					1.000	
Currently married					0.500	[0.16-1.55]
Formerly married					0.607	[0.30-1.22]
**Education level**						
None					1.000	
Primary					1.795	[0.87-3.71]
Secondary or higher					2.128*	[1.01-4.47]
**Wealth index**						
Poorest					1.000	
Poorer					1.077	[0.68-1.71]
Middle					1.519	[0.95-2.44]
Richer					2.518^***^	[1.58-4.01]
Richest					2.466**	[1.44-4.22]
**Children surviving**						
None					1.000	
1-4					2.039*	[1.16-3.59]
5+					1.655	[0.85-3.21]
**Fertility preference**						
Have another child					1.000	
Undecided					0.916	[0.30-2.81]
No more					1.166	[0.81-1.69]
No stable partner/sterilized/infertile					3.503*	[1.28-9.61]
**Observations**	**1755**		**1755**		**1755**	

^*^p < 0.05,^**^p < 0.01,^***^p < 0.001; CI: Confidence Intervals; OR: Odds Ratios; Model 2 – adjusting for demographics, Model 3 – adjusting for demographics and socioeconomic factors.

Discussing family planning with a health worker was significantly associated with use of modern contraceptives (OR = 1.64; 95% CI: 1.19–2.26) in the unadjusted analyses as presented in Model 1. The relationship persisted (OR = 1.85; 95% CI: 1.29–2.66) even after adjusting for behavioral, demographic and socioeconomic variables in Model 3.

Subsequent to adjusting for possible confounding, region (southwest), education level (secondary or higher), wealth quintile (richer and richest), children surviving (1–4) and fertility preferences (no partner) were found to be significantly associated with men’s reported use of contraception and men’s reporting their partner’s use of contraception. Men from the southwest region had a lower likelihood of using or reporting their partners’ use of modern contraceptives (OR =0.41; 95% CI: 0.21–0.77) compared with men in Kampala. In addition, men who had secondary or higher education had increased odds (OR =2.13; 95% CI: 1.01–4.47) of MCU compared with those with no education. Men who belonged to the richer and richest wealth quintile had a higher likelihood of using modern contraceptives (OR =2.52; 95% CI: 1.58–4.01) and (OR =2.47; 95% CI: 1.44–4.22) respectively compared with those in the poorest quintiles. Men who had few children (1–4) had increased odds of using modern contraception or reporting partners’ use of contraception (OR = 2.039; 95% CI: 1.16–3.59) compared with those with no children. Additionally, men who had no partner and were never married had a higher likelihood of using modern contraception or reporting partners’ use of contraception (OR = 3.50; 95% CI: 1.28–9.61) compared with those who wanted to have additional children (see Model 3 in Table 3).

In Model 2, men’s age and residence were significantly associated with use of modern contraceptives. However, after adjusting for confounders (Model 3), these associations became insignificant (p > 0.05).

## Discussion

The results presented illuminate the critical role of interpersonal communication, and particularly discussion of FP issues involving men and health workers in effecting behavior change. Male respondents who had discussed family planning with a health worker were more likely to use modern contraceptives than those who had not. Discussion of family planning with health workers improves client’s knowledge of contraception and therefore, contributes to beneficial behavior change. Behavior change models stipulate that knowledge is a primary step toward achieving behavior change [34]. This finding is in keeping with findings from a study conducted in Congo where current use of modern contraception was correlated with having discussed contraception with a health worker [35]. Similar results were also reported in a Tanzanian study where training of service providers, communication, and logistical support influenced MCU [36].

Our study further showed that the number of living children was an important factor influencing the use of modern contraception. The men who had fewer than five children had increased odds of using contraception. This is an important finding because although numerous demographic studies on women have indicated this relationship [37,38], little is known about child survival status and men’s preference for more children [39].

Socioeconomic status is closely linked with people’s behavior and practices, and contraceptive use in particular. In this paper, men from the southwest region were less likely to use modern contraceptives compared with those from Kampala. Over the past decades in Uganda, the western region has recorded the highest fertility rates [40,41]. According to JP Ntozi [5], the western region has a pro-natalist culture which discourages contraceptive use until a woman has six to eight live children, including at least two sons. Perhaps this explains why men in this region are less likely to use modern contraception. Some studies have indicated that contraceptive use varies across regions and cultural environment [41,42].

As observed elsewhere [43-45], this study established that wealth status and education level were key determinants of men’s modern contraceptive use. For instance, the likelihood of contraceptive use was higher among rich men with post primary education compared to those men in lowest wealth quintile with nor formal education.

Our findings show that men without partners or who were sterilized/infertile had higher odds of reporting modern contraceptive use compared with those who wanted more children. The sexually active men who had no partners were also the never married ones. Therefore, they used modern contraceptives as a means of preventing pregnancy in the context of unstable relationships where child bearing is not ideal. In such a context, these men preferred to delay fathering children [46].

Some limitations of the paper are worth highlighting. First, the use of the DHS data poses a challenge to establishing the direction of causality. This is particularly so with regard to discussion of FP with a health worker as a predictor of MCU, given the cross-sectional nature of the survey. The authors assumed that discussing family planning with a health worker prompted contraceptive use but the reverse could also have been true. Using contraception may have prompted discussion of contraception with a health worker. It is also possible that men who wanted to use contraception methods were more likely to talk to a provider. Therefore more provider communication would do not impact the men who do not seek to discuss with providers because they have no interest. There could also have been other factors that may have influenced MCU by the men and their reportage of partners’ use, other than discussion.

Second, there was no provision in the dataset to ascertain the context, content, or depth of the discussions between the health workers and their clients (the men). This information would have further enriched our analyses. However, despite these limitations, the paper provides an interesting contribution to the debate on male involvement in family planning, which has been a neglected issue for some time in the Ugandan context.

## Conclusions

Our findings confirm the strong association that discussion with a health worker has on the likelihood of men’s use of modern contraception and their of partners’ use of contraception. Fertility preference, number of surviving children, wealth index, level of education and region were also among the sociodemographic predictors of MCU. Health workers should therefore be targeted as focal persons in delivering FP messages to men. FP programs should be designed and tailored to enhance FP among men with more than four children and those with low socioeconomic status i.e. the poor and those with low levels of education.

## Abbreviations

FP: Family planning; DHS: Demographic and health survey; MCU: Modern contraceptive use; UDHS: Uganda demographic and health survey; UBOS: Uganda Bureau of Statistics.

## Competing interests

The authors declare that they have no competing interests.

## Authors’ contributions

AK conceived the study, participated in data analyses, scientific content, interpretation of findings, discussion, conclusions and writing the manuscript. PN conceived the study, participated in background write up, interpretation and in manuscript write up. SOW participated in data analysis and review of manuscript, and BK participated in manuscript review, discussion and interpretation of results. All authors read and approved the final manuscript.

## Pre-publication history

The pre-publication history for this paper can be accessed here:

http://www.biomedcentral.com/1471-2458/14/286/prepub

## Supplementary Material

Additional file 1 DHS questions asked to men about current use of contraceptives. Click here for file
