# Supplementary material for: Modern contraceptive use among sexually active men in Uganda: does discussion with a health worker matter?
Source: BMC Public Health. 2014 Mar 28;14:286. doi: 10.1186/1471-2458-14-286 (PMC3986853; doi:10.1186/1471-2458-14-286)
Supplement: Additional file 1 — DHS questions asked to men about current use of contraceptives. [file 1471-2458-14-286-S1.docx]

# Additional file 1

### Question and filters asked to men for use of contraception

**MV312** which is current use of modern contraception is generated based on two questions in men’s questionnaire- Q438 and Q439 as shown in the question and filters below.


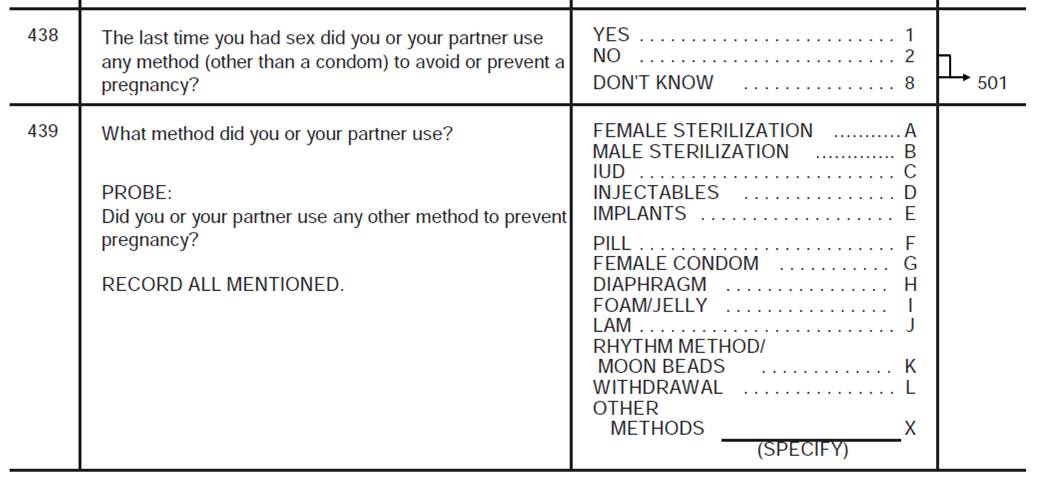


The above questions were used to generate the dependent variable- modern contraceptive use.
